# Supplementary material for: The cost of inaction to strengthen the resilience of primary health care in Latin America and the Caribbean: a modelling study
Source: Lancet Reg Health Am. 2025 Sep 29;50:101248. doi: 10.1016/j.lana.2025.101248 (PMC12541822; doi:10.1016/j.lana.2025.101248)
Supplement: Supplementary Appendix [file mmc2.pdf]

Appendices

Appendix A: Data availability for countries across different service domains

Table A.1: Service coverage data availability across countries

ISO	Family planning	ANC visits	Pregnancy related routines and maternal nutrition	Vaccines	Child health	NCD
ARG	✓	✓	X	✓	X	✓
ATG	X	X	X	X	X	✓
BHS	✓	✓	X	✓	X	✓
BLZ	✓	✓	✓	✓	✓	✓
BOL	✓	✓	✓	✓	✓	✓
BRA	✓	✓	✓	✓	X	✓
BRB	X	X	X	X	X	✓
CHL	✓	✓	X	✓	X	✓
COL	✓	✓	✓	✓	X	✓
CRI	✓	✓	X	✓	X	✓
CUB	✓	✓	X	✓	X	✓
DMA	X	X	X	X	X	✓
DOM	✓	✓	✓	✓	✓	✓
ECU	✓	✓	✓	✓	X	✓
GRD	✓	✓	X	✓	X	✓
GTM	✓	✓	✓	✓	✓	✓
GUY	✓	✓	✓	✓	X	✓
HND	✓	✓	✓	✓	✓	✓
HTI	✓	✓	✓	✓	✓	✓
JAM	✓	✓	X	✓	X	✓
KNA	X	X	X	X	X	✓
LCA	✓	✓	X	✓	X	✓
MEX	✓	✓	✓	✓	✓	✓
NIC	✓	✓	✓	✓	✓	✓
PAN	✓	✓	X	✓	✓	✓
PER	✓	✓	✓	✓	✓	✓
PRY	✓	✓	X	✓	✓	✓
SLV	✓	✓	✓	✓	✓	✓
SUR	✓	✓	X	✓	X	✓
TTO	✓	✓	X	✓	X	✓
URY	✓	✓	X	✓	X	✓
VCT	✓	✓	X	✓	X	✓
VEN	✓	✓	✓	✓	X	✓

Appendix B: Additional modelling details

B.1. Interventions and NCDs included in the modelling

Table B.1: Interventions considered in LiST based model

Category	Intervention
**Antenatal Care**	ANC (1 visit)
	ANC (>4 visits)
	Tetanus toxoid vaccination
	Prevention of malaria during pregnancy
	Syphilis detection and treatment*
	Iron supplementation
	Multiple micronutrient supplementation
	Hypertensive disorder case management*
	Diabetes case management*
	Malaria case management*
**Vaccines**	Pentavalent (DPT, Hib, HBV)
	Pneumococcal - Three doses
	Rotavirus – three doses
	Meningococcal A single dose
	Malaria vaccine booster
	Measles single dose
	Measles two doses
**Child health**	Vitamin A supplementation
	Zinc supplementation

*Coverage for these pregnancy interventions are calculated in LiST as proportional to ANC utilization multiplied by an estimate for the quality of ANC (refer appendix C)

Table B.2: NCD conditions considered for modelling

Cardiovascular disease	Diabetes	Chronic respiratory disease	Mental disorders
• Rheumatic heart disease<br>• Ischemic heart disease<br>• Stroke<br>• Hypertensive heart disorder<br>• Non-rheum valve diseases<br>• Cardiomyopathy<br>• Pulmonary arterial hypertension<br>• Atrial fibrillation<br>• Peripheral artery<br>• Endocarditis<br>• Other cardiovascular	• Diabetes mellitus type 2	• Chronic obstructive pulmonary disease	• Any psychological disorder

B.2. NCD model

B.2.1. NCD model: baseline projections

The NCD model is a simple compartmental model that tracks the population of each country over time. For each country (*c*), the model includes a set of cause-specific mortality rates (age- and sex-standardised) for each of the conditions (*i*) in Table B.2, in the year *t*. These are denoted

$$\mu_{i,t}^{c}$$

If P_t^c is the number of people in country *c*, in year *t*, then the number of deaths attributable to cause *i* in the year *t* is given by

$$P_t^c * \mu_{i,t}^{c}$$

The values of P_t^c are provided in Appendix D, Table D.1 (extracted from World Population Prospectus 2024, projected incorporating fertility and overall mortality)[1]. The values for $\mu_{i,t}^{c}$ for the most recent year (2024) are taken from the Institute of Health Metrics and Evaluation (Appendix D, Table D.5) and assumed to remain constant over time in the absence of changes to intervention coverages.

B.2.2. NCD model: interventions

The cause-specific mortality rates in the model can be influenced by the coverage of primary health care services. Let $COV_{i,t}^{c}$ be the proportion of people in country *c* with condition *i* in year t who have their condition controlled, and RR_i be the relative risk of mortality from condition *i* when it is controlled compared to not controlled. Then

$$\mu_{i,t}^{c} = \widehat{\mu_{i,t}^{c}} * \left[RR_i * COV_{i,t}^{c} + \left(1 - COV_{i,t}^{c}\right)\right]$$

where $\widehat{\mu_{i,t}^{c}}$ is the reconciled mortality rate in the absence of any of the conditions in Table B.2 being controlled. The reconciled mortality rate is calculated in the model on initialisation based on inputs for baseline control levels (Appendix D, Table D.6), relative risks (main paper Table 1), and baseline mortality rates (Appendix D, Table D.5).

Once $\widehat{\mu_t^c}$ has been calculated, the following formula is used to calculate cause-specific deaths ($D_{i,t}^{c}$) when the proportion of people with each condition under control is varied

$$D_{i,t}^{c} = P_t^c * \widehat{\mu_{i,t}^{c}} * \left[RR_i * COV_{i,t}^{c} + \left(1 - COV_{i,t}^{c}\right)\right]$$

B.2.3. NCD model: shock scenarios

Depending on the magnitude and recovery duration of the shock, the proportion of people with controlled NCDs ($COV_{i,t}^{c}$) over time during a disruption event is derived using

$$COV_{i,t}^{c} = COV_{i,0}^{c} * (1 - M_t)$$

where $COV_{i,0}^{c}$ is the initial proportion under control and M_t is the relative size of the disruption in year t (see appendix Table D.6 for data inputs for $COV_{i,0}^{c}$).

This disruption impact is taken from the following shock scenarios:

- **Short PHC shock (low):** $M_t = 0.25$ if $t = 2026$ and $M_t = 0$ for all $t \in$ [2027, 2030]
- **Short PHC shock (high):** $M_t = 0.50$ if $t = 2026$ and $M_t = 0$ for all $t \in$ [2027, 2030]
- **Long PHC shock (low):** $M_t = 0.25$ if $t = 2026$ and M_t linearly decreased to zero for $t \in$ [2027, 2030]
- **Long PHC shock (high):** $M_t = 0.50$ if $t = 2026$ and M_t linearly decreased to zero for $t \in$ [2027, 2030]

B.3. Family planning model

B.3.1. Family planning model overview

The family planning model is a simple compartmental model that tracks the number of women of reproductive age in each country over time (estimated 15-49-years-olds, extracted from world population prospects 2024, Table D.2). Let

- $P_t^{w,c}$ be the population size of the women 15-49 years in country c and year t;
- U_t^c be the unmet need for family planning among women 15-49 years in year t (Table D.3);
- $CPR_t^{M,c}$ be the proportion of women 15-49 years in country c using modern contraception methods in year *t*;
- $CPR_t^{T,c}$ be the proportion of women 15-49 years in country c using traditional contraception methods in year *t;*
- $f^{M,c}$ be the weighted-average modern contraception failure rate in country c (Table D.3);
- $f^{T,c}$ be the failure rate of traditional contraception methods in country c (18%; assumed constant across the countries modelled).
- F_t^c be the average pregnancy rate among women 15-49 years in country c and year t

Then for each country *c*, the number of pregnancies in year *t* is obtained using

$$P_t^{w,c} * F_t^c$$

The pregnancy rate F_t^c can be deconstructed as

$$F_t^c = \widehat{F^c} * \left(U_t^c + CPR_t^{M,c} * f^{M,c} + CPR_t^{T,c} * f^{T,c}\right)$$

where $\widehat{F^c}$ is the reconciled pregnancy rate among women with unmet need for contraception. Note that $U_t^c + CPR_t^{M,c} + CPR_t^{T,c} \leq 1$ by definition. The reconciled pregnancy rate is calculated in the model on initialisation based on inputs for baseline unmet need for contraception, baseline contraception prevalence rates and baseline pregnancy rates among women of reproductive age (for this analysis birth rates were used as a proxy for pregnancy rates; Table D.3).

B.3.2. Family planning model: shock scenarios

The impact of health system shocks on family planning services was modelled as a relative reduction in the proportion of women accessing modern contraception services in each country *c* in year *t* ($CPR_t^{M,c}$). Those impacted were modelled to shift from modern methods (average effectiveness 91-99% based on country-specific methods mix; Table D.3) to traditional methods (average effectiveness of 82%) leading to increased proportion of women who use traditional methods of contraception in each country in year *t* ($CPR_t^{T,c}$).

$$CPR_t^{M,c} = CPR_0^{M,c} * (1 - M_t)$$

$$CPR_t^{T,c} = CPR_0^{T,c} + CPR_0^{M,c} * M_t$$

where $CPR_0^{M,c}$ and $CPR_0^{T,c}$ are the initial proportion of women 15-49 years in country c using modern and traditional contraception methods, respectively, and M_t is the relative size of the disruption in year t.

This disruption impact for the different shock scenarios is as per section B.2.3 above.

Appendix C: Estimating the coverage based on utilization and quality: LiST

Ten antenatal care interventions were included in this study from LiST (Table B.1). Of these, Syphilis detection and treatment, hypertensive disorders case management, diabetes case management and malaria case management do not have data on coverage from household surveys. The coverages of these interventions are estimated using service utilization and quality (Table C.1).

Table C.1. Antenatal care interventions estimated from utilization and quality

Intervention	Default data source for coverage
Syphilis detection and treatment	Calculated from utilization (at least 1 ANC visit) and quality
Hypertensive disorders case management	Calculated from utilization (at least 4 ANC visit) and quality
Diabetes case management	Calculated from utilization (at least 4 ANC visit) and quality
Malaria case management	Calculated from utilization (at least 4 ANC visit) and quality

Quality/readiness-adjusted coverage

For the interventions in Table C.1, LiST estimates coverage by multiplying utilization by quality/readiness of clinics to provide that service:

$$\text{Coverage estimates} = \text{Quality} \times \text{Utilization}$$

For antenatal care interventions, utilization is based on the attendance to antenatal care clinics (either at least 1 ANC visit, or at least 4 ANC visits; Table C.1). Major household surveys conducted in countries are used to measure the utilization matrices of antenatal care (e.g. DHS, MICS). Quality is a consideration that impacts overall coverage, modifying for the proportion of women who visit antenatal care. Facility survey programs such as Service Provision Assessments (SPA) and Service Availability and Readiness Assessment (SARA) collect data on the services different health facilities are able to provide.[2] In these surveys, drugs, supplies equipment and tests available at the clinics are recorded in addition to the checks on training and supervision of service providers.[2, 3]

Appendix D: Data inputs and model outputs

See supplementary Excel file.

Table D.1: Population projections for countries in the LAC (Totals)

Table D.2: Population estimations for countries in the LAC (for age bins; by sex)

Table D.3: Epidemiological indicator data

Table D.4: Prevalence rates of NCD conditions

Table D.5: Mortality rates for NCD conditions

Table D.6: Percentage of population controlled their NCD conditions (estimated)

Table D.7: Death distribution of NCDs according to five-year age bins

Table D.8: Expected healthy life years for countries in LAC (discounted)

Table D.9: Expected healthy life years for countries in LAC (undiscounted)

Table D.10: Parameters for the economic evaluations

Table D.11: Percentage of ANC utilization and coverages of interventions (pregnancy, child health and vaccines) – Baseline

Table D.12: Percentage of ANC utilization and coverages of interventions (pregnancy, child health and vaccines) – short (low)

Table D.13: Percentage of ANC utilization and coverages of interventions (pregnancy, child health and vaccines) – short (high)

Table D.14: Percentage of ANC utilization and coverages of interventions (pregnancy, child health and vaccines) – long (low)

Table D.15: Percentage of ANC utilization and coverages of interventions (pregnancy, child health and vaccines) – long (high)

Table D.16: Percentage of women (18-49 years old) use modern contraception methods (CPR modern) for shock scenarios

Table D.17: Table D.17: Percentage of women (18-49 years old) use traditional contraception methods (CPR traditional) for shock scenarios

Table D.18: Modelled cause specific mortality rates (NCDs) for scenarios

Table D.19: Modelled cause specific number of deaths (due to NCDs) for scenarios

Table D.20: Proportion of the population controlled NCDs for scenarios

Appendix E: Economic model description

The economic cost of shocks to primary health care service coverage (antenatal care, prevention, family planning services and management of non-communicable diseases) were considered across two domains:

1. **Social**: increased mortality (increased maternal deaths, stillbirths, neonatal deaths and child deaths) and increased morbidity (years of life with disability due to uncontrolled non-communicable diseases) leading to economic costs
2. **Workforce participation**: reduced workforce participation due to unintended pregnancies

Economic costs of primary health care shocks were calculated based on the difference in outcomes between the strengthened scenario (assuming no shocks to primary health services delivery) compared with each health system shock scenario specified by its duration and the intensity.

Social costs from years of life lost

The Lives Saved Tool (LiST) was used to model reduced coverage of antenatal care interventions due to shocks and calculate the additional number of maternal deaths, stillbirths, neonatal deaths and child deaths for each year. Decreased coverage of family planning was also modelled separately, resulting in an increased number of unintended pregnancies as well as additional maternal deaths, which were calculated according to the maternal mortality rates among current pregnancies for each country.

Additional maternal deaths, stillbirths, neonatal deaths and child deaths were converted to total years of life lost using country- and age-specific distributions of expected healthy life years remaining (Table D.8). Stillbirths and neonatal deaths were assumed to have years of life loss equivalent to the expected number of healthy life years of newborns and child deaths were assumed to have years of life loss equivalent to the expected number of healthy life years of a child less than five years old. Country-specific distributions for the age of pregnancies were used to estimate the age of maternal deaths and corresponding expected number of healthy life years lost. There is some debate about years of life gained from averting stillbirths (more specifically disability-adjusted life years gained)[4], and for this analysis years of life lost were considered for 19.7% of the stillbirths occurred, which is the estimate for the Latin America region that are intrapartum[5].

Excess deaths from non-communicable disease due to reduced management during shocks were also converted to years of life lost, based on country-specific age distributions of deaths from each condition included in the analysis (Table B.2). More specifically, for each country (c), the healthy life years lost in year t due to all conditions (i) in Table B.2, YLL_t^c was calculated as

$$YLL_t^c = \sum_i D_{i,t}^c \sum_a p_{i,a}^c L_a^c$$

where $D_{i,t}^{c}$ is the number of cause-specific deaths in country c, from condition i in year t, (see appendix B), $p_{i,a}^{c}$ is the proportion of deaths from condition i in each age group a (Table D.7), and L_a is the discounted expected healthy life years of individuals in the age category a (see appendix Table D.8 for undiscounted and Table D.9 for discounted data inputs).

Since the shocks were modelled as short duration, the same values of country- and age-specific expected healthy life years remaining were used for each year of the model projections.

GDP per capita for each country (see appendix Table D.10) was used to convert years of life lost into economic costs, with 3% per annum discounting applied to future years.

Social costs from years lived with disability

The main analysis of this study did not consider years lived with disability. However, in a sensitivity analyses they were considered: a prevalence-based approach was used to calculate the years lived with disability[6], assuming that people who access primary health care have reduced disutility weights for each NCD. Due to primary health care shocks, a greater proportion of people would move to experiencing the higher disutility weight, leading to an increase in total years lived with disability.

Costs of reduced workforce participation due to unintended pregnancies

Additional unintended pregnancies occurring among women >18 years due to primary health care shocks were assumed to lead to a reduction in workforce participation. Pregnancy was assumed to remove a woman from the labour force for several months, based on country-specific maternity leave policies (Table D.10) and adjusted for country specific workforce participation rates among women.

The economic cost was then calculated as the duration of maternity leave multiplied by the GDP per worker.

Appendix F: Additional results

Total outcomes

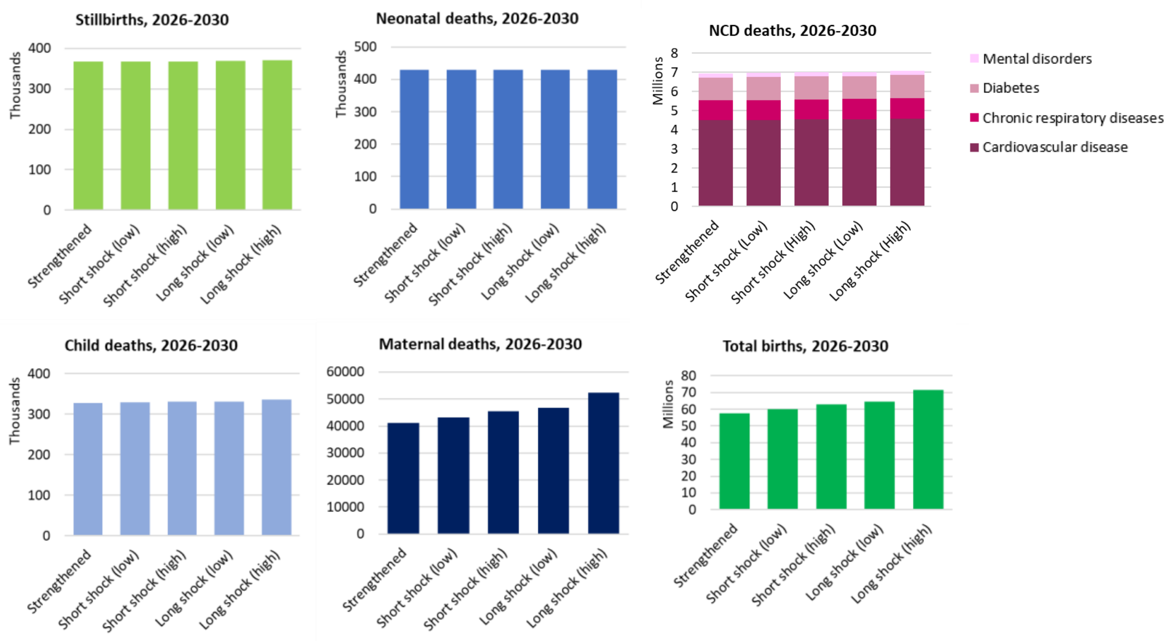

Figure F.1: Cumulative total health outcomes for each shock scenario compared with "strengthened" scenario. Total stillbirths (top; left), neonatal deaths (top, middle), NCD deaths (top; right), child deaths (bottom; left), maternal deaths (bottom; middle) and births (bottom; right) over 2026-2030

Sensitivity analysis: including stillbirths and neonatal deaths due to shocks in family planning services

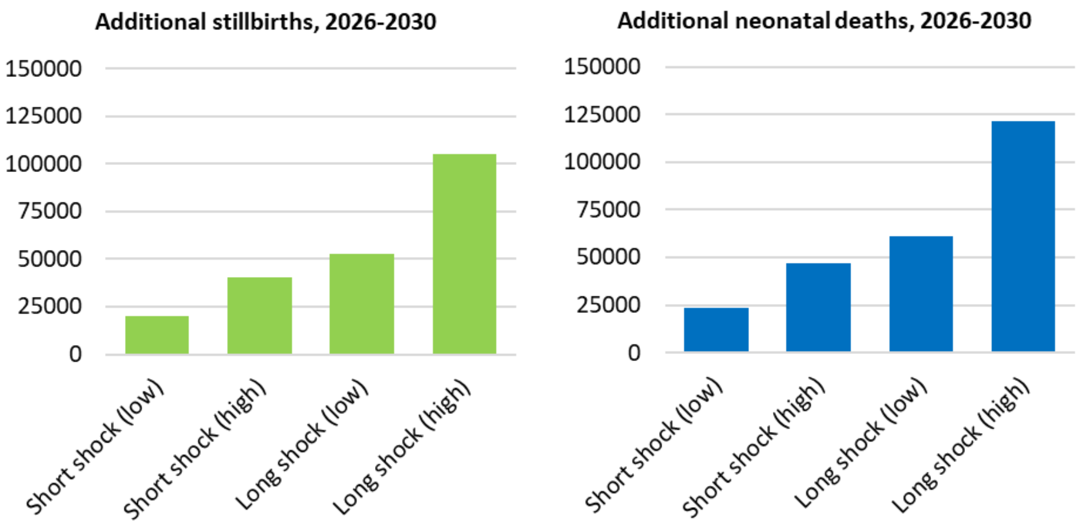

Figure F.2: Cumulative stillbirths (left) and neonatal deaths (right) due to shocks in maternal health as well as family planning services

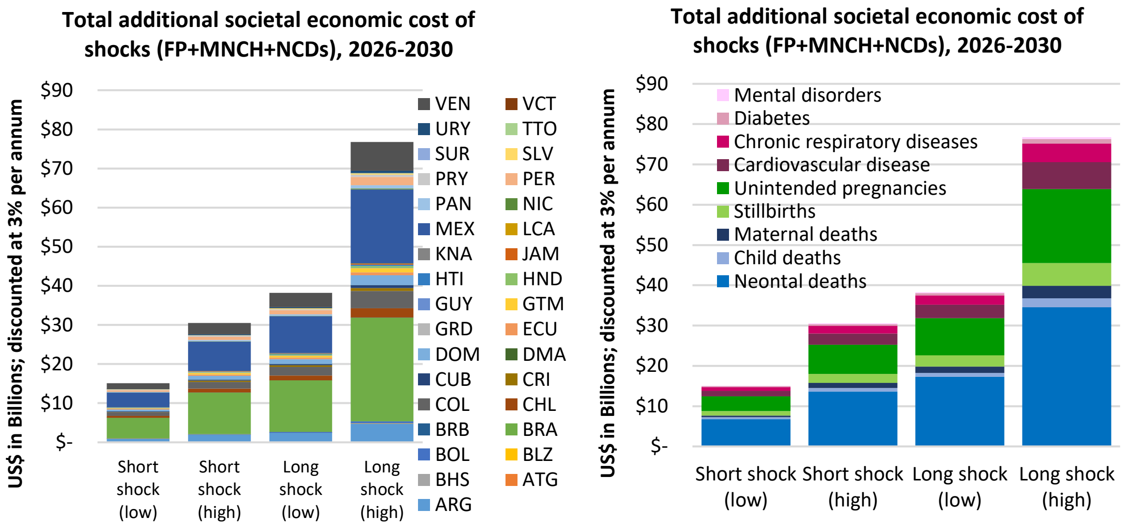

Figure F.3: Cumulative societal economic cost of primary health care shock scenarios, disaggregated across countries (left), and across cause of mortality or unintended pregnancies (right). **In this figure, the impact of shocks to family planning services on stillbirths and neonatal deaths is also included.** Costs are total over 2026-2030 across the 33 countries and subsets of service areas modelled, presented in 2023 US$ with 3% per annum discounting applied. The economic costs vary across countries due to relative population size and epidemiological indicators. FP, family planning; MNCH, maternal, newborn and child health; NCDs, non-communicable diseases.

<u>*Sensitivity analysis: including a 5% reduction in disutility weight when accessing primary health care*</u>

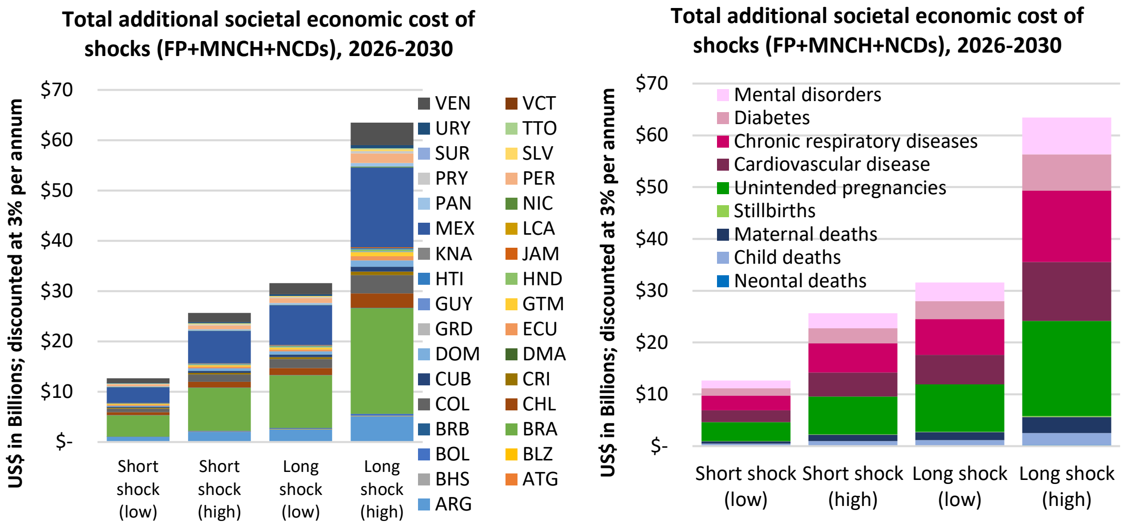

Figure F.4: Cumulative societal economic cost of primary health care shock scenarios, disaggregated across countries (left), and across cause of mortality or unintended pregnancies (right). **An assumed 5% reduction in disutility when NCD conditions are controlled included**. Costs are total over 2026-2030 across the 33 countries and subsets of service areas modelled, presented in 2023 US$ with 3% per annum discounting applied. The economic cost**s** vary across countries due to relative population size and epidemiological indicators. FP, family planning; MNCH, maternal, newborn and child health; NCDs, non-communicable diseases

Additional figures

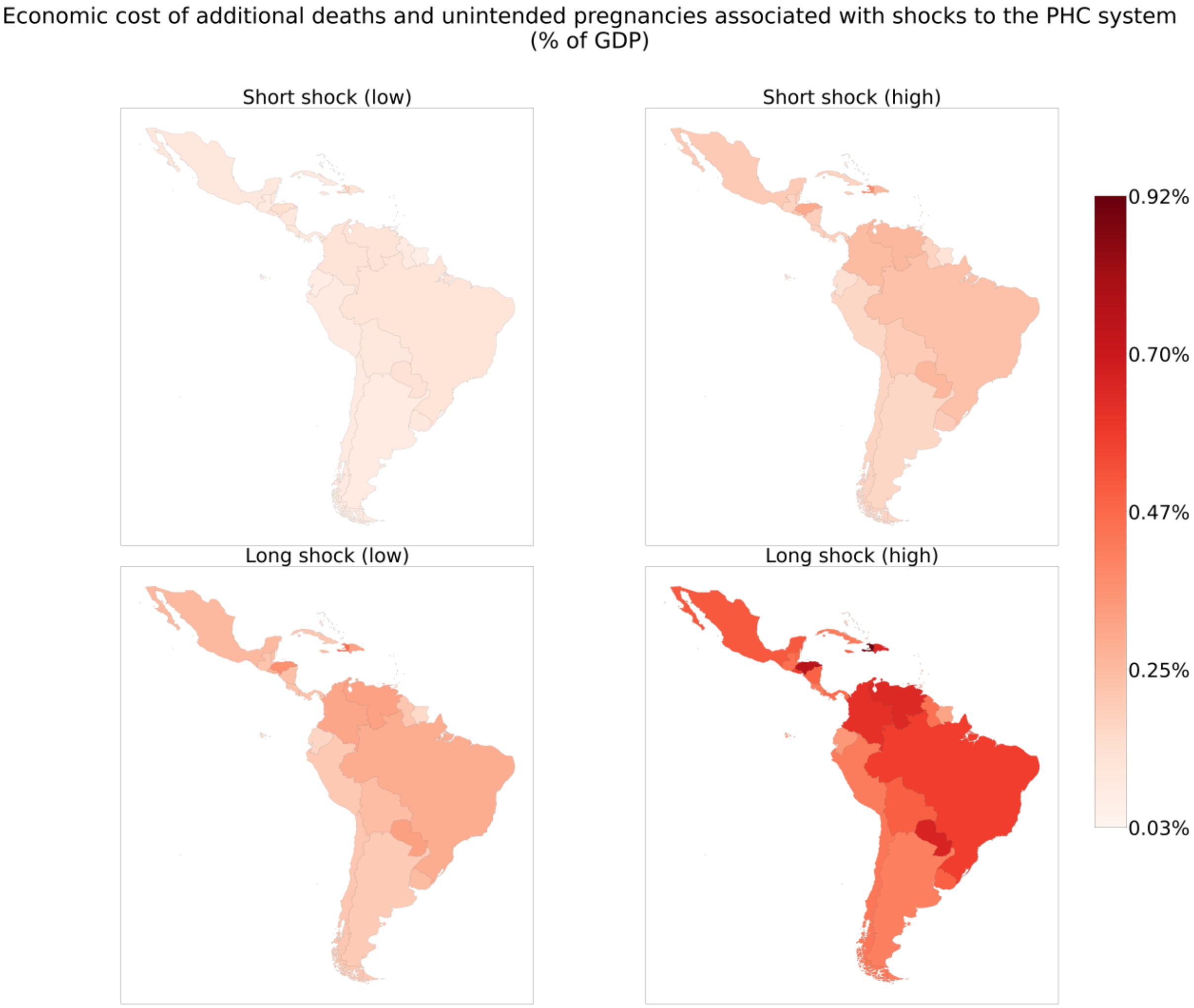

Figure F.5: Geographic differences of economic cost of primary health care shocks as a percentage of the national GDP across the countries in LAC. 2023 GDP values were used; the economic cost in some countries may appear small relative to population size as not all intervention categories could be included for some of the smaller countries (Appendix A). The economic costs of the shocks are spread over many years but totals are shown as a percentage of annual GDP for contextualisation.

Supplementary references

1. World Population Prospects 2024 [Internet]. 2024. Available from: https://population.un.org/wpp/.
2. Spectrum. Lives Saved Tool Technical Note2022. Available from: https://www.livessavedtool.org/technical-notes.
3. Avenir Health. Spectrum Manual Spectrum System of Policy Models 2024. Available from: https://avenirhealth.org/Download/Spectrum/Manuals/SpectrumManualE.pdf.
4. Jamison DT, Shahid-Salles SA, Jamison J, Lawn JE, Zupan J. Incorporating deaths near the time of birth into estimates of the global burden of disease. Global burden of disease and risk factors: The International Bank for Reconstruction and Development/The World Bank; 2006.
5. de Mucio B, Sosa C, Colomar M, Mainero L, Cruz CM, LM Cv. The burden of stillbirths in low resource settings in Latin America: Evidence from a network using an electronic surveillance system. PLoS ONE 2023;18(12):e0296002.
6. WHO. WHO methods and data sources for global burden of disease estimates 2000-2019. In: WHO, editor. Department of Data and Analytics Division of Data, Analytics and Delivery for Impact WHO, Geneva2020.
